# Optimizing NK Cell-Based Immunotherapy in Myeloid Leukemia: Abrogating an Immunosuppressive Microenvironment

**DOI:** 10.3389/fimmu.2021.683381

**Published:** 2021-06-17

**Authors:** Natasha Mupeta Kaweme, Fuling Zhou

**Affiliations:** Department of Hematology, Zhongnan Hospital, Wuhan University, Wuhan, China

**Keywords:** acute myeloid leukemia, natural killer cells, immunotherapy, immunosuppressive microenvironment, enhancing strategies

## Abstract

Natural killer (NK) cells are prominent cytotoxic and cytokine-producing components of the innate immune system representing crucial effector cells in cancer immunotherapy. Presently, various NK cell-based immunotherapies have contributed to the substantial improvement in the reconstitution of NK cells against advanced-staged and high-risk AML. Various NK cell sources, including haploidentical NK cells, adaptive NK cells, umbilical cord blood NK cells, stem cell-derived NK cells, chimeric antigen receptor NK cells, cytokine-induced memory-like NK cells, and NK cell lines have been identified. Devising innovative approaches to improve the generation of therapeutic NK cells from the aforementioned sources is likely to enhance NK cell expansion and activation, stimulate *ex vivo* and *in vivo* persistence of NK cells and improve conventional treatment response of myeloid leukemia. The tumor-promoting properties of the tumor microenvironment and downmodulation of NK cellular metabolic activity in solid tumors and hematological malignancies constitute a significant impediment in enhancing the anti-tumor effects of NK cells. In this review, we discuss the current NK cell sources, highlight ongoing interventions in enhancing NK cell function, and outline novel strategies to circumvent immunosuppressive factors in the tumor microenvironment to improve the efficacy of NK cell-based immunotherapy and expand their future success in treating myeloid leukemia.

## Highlights

- NK cell-based immunotherapy continues to expand in clinical practice.
- Malignantly transformed cells and tumor progression lead to an immunosuppressed microenvironment, limiting the efficacy of various immunotherapies.
- Strategizing methods to enhance NK cell expansion, *in vivo* persistence and cytotoxicity may improve the treatment response of conventional chemotherapy.

## Introduction

Acute myeloid leukemia (AML) is an aggressive malignant disease of high heterogeneity that remains a deterring challenge to clinicians due to shortened remission duration and high relapse rates associated with the disease. Advanced sequencing technologies have revealed the impact of several aberrant chromosomes, genomic mutations, and myeloid transcription factor dysregulation on the pathogenesis of AML (1). Over the past 30 years, the treatment of AML has not invoked drastic evolvement, with the standard of treatment being chemotherapy. Chemotherapy employs the pro-oxidant approach to induce apoptosis but causes high toxicity, chemo-refractory and disease relapse, resulting in increased morbidity, and mortality of patients with AML (2).

Chronic myeloid leukemia (CML) is a myeloproliferative malignancy genetically characterized by a translocation (3, 4) in the hematopoietic stem cell resulting in constitutively active BCR-ABL1 oncokinase. The majority of patients present with abnormal immune cells (5). On the basis of the critical role of BCR-ABL1 oncokinase activity, tyrosine kinase inhibitors (TKIs) remain the standard therapy (6, 7). Although targeted therapy such as imatinib effectively induce and sustain remissions, molecular disease often persists after years of treatment (8, 9). Mahon et al. showed that imatinib could be discontinued without relapse in only a subset of patients, indicating a possibility of a cure with TKIs (10).

In recent years, the pullulating of novel anticancer therapies to enhance immune response has pushed natural killer (NK) cells into the spotlight (3, 11). NK cells are crucial cytotoxic and cytokine-producing components of the innate immune system that represent crucial effector cells in cancer immunotherapy. As an integral part of the innate lymphoid cells (ILCs), NK cells play vital roles in antiviral and anti-tumor defense (12). ILCs are a heterogenous group of immune cells that maintain homeostasis and respond to pathogen invasion through an immune-mediated response (13). NK cells are derived from common lymphoid progenitors differently from T and B cells and do not express antigen-specific receptors. However, NK cells express a remarkedly wide array of germline-encoded inhibitory and activating receptors on their surface, mediating immune response (14).

NK cell-based anticancer therapies have been highly exploited in hematological malignancies in present-day practice (15). In acute leukemia, particularly in children given human leucocyte antigen (HLA)-haploidentical hematopoietic transplantation, infusion of mature NK cells through graft manipulation based on the selective depletion of T cells and CD19+B cells improved outcome (16–18). An increased proportion of mature NK cells was associated with molecular relapse-free survival in CML patients. Ilander et al. showed that NK cells were important in sustaining remission with imatinib discontinuation (8, 19). Similarly, Dasatinib discontinuation in patients with CML who maintained deep molecular response was successful in 50% of patients (20). In the IMMUNOSTIM study, non-relapsing patients had higher numbers of NK cells of the CD56^dim^ subset than relapsing patients (21).

In the recent past, strategies to optimize NK cell function have aimed at improving NK cell targeting, anti-tumor response, and limiting NK cell inhibition. Various sources of NK cells such as chimeric antigen receptor (CAR) NK cells, NK cells derived from haploidentical donors (i.e. having half HLA matched), stem cell-derived NK cells, NK cell lines, umbilical cord blood (UCB) NK cells, adaptive NK cells, and cytokine-induced memory-like (CIML) NK cells have been exploited in clinical practice (22). Enhancement of NK cell generation from various sources and modulation of NK cell expansion, cytotoxicity, and *in vivo* survival have changed the treatment paradigm for AML.

Despite breakthroughs in novel NK cell sources, NK cells remain subject to prominent immunosuppressive barriers present in the tumor microenvironment (TME), resulting in the dysfunction and exhaustion of NK cells. Devising methods to overcome these immunosuppressive factors to enhance NK cell function and achieve the desired outcome from therapies is imperative (22). In this review, we discuss the current NK cell sources, highlight ongoing interventions in enhancing NK cell function, and outline novel strategies to circumvent immunosuppressive factors in the TME to improve the efficacy of NK cell therapy and expand their success in future research.

## NK Cells in Myeloid Leukemia

NK cells express rapid and efficient cytolytic activity in recognizing and killing both transformed and virally infected cells. Through the release of cytoplasmic cytotoxic granules containing perforin, granulysin, and granzymes A and B (4), or by using effector molecules such as tumor necrosis factor, tumor-related apoptosis-inducing ligand (TRAIL), and Fas ligand, NK cells can induce apoptosis to target cells. NK cell activation in the host promotes pro-inflammatory cytokine production that affects both hematopoietic and non-hematopoietic cell function (23). Through secretion of cytokines and chemokines and direct cell-to-cell contact, NK cells promote activation and effector functions of other innate and adaptive immune cells contributing to homeostasis of the immune system. NK cells express activating and inhibitory receptors, that distinguish normal from transformed cells and recognize altered protein expression on target cells, thereby controlling the cytolytic function (24).

Evidence shows that NK cells can exhibit anti-tumor activity against leukemic blasts from patients with AML, CML, or MDS. More recent studies have mainly focused on exploiting NK cells to target AML. Cytokine-mediated activation of NK cells with IL-2 or IL-15 is commonly employed and is currently extensively studied (25). In recent investigations, the pre-activation of mouse and human NK cells with a cocktail of IL-12/15/18 (26) had enhanced and sustained anti-tumor functions *in vitro* and *in vivo* after infusion. Similarly, Romee et al. reported that CIML NK cells exhibited enhanced antileukemia functionality and that human memory-like NK cells had enhanced interferon-γ (IFN-γ) production and cytotoxicity against leukemia cell lines or primary human AML blasts *in vitro* (27, 28).

Additional studies investigated the beneficial effect of *ex-vivo* expanded NK cells against well-established cell lines such as K562 cells (27, 29). Hasmim and colleagues further investigated the *in vivo* anti-leukemic capacity of *ex vivo*-expanded NK cells against patient-derived AML cells. The study concluded that the surface expression of CD94 on *ex vivo*-differentiated NK cells was a potential indicator of *in vitro* and *in vivo* killer cell functionality (30).

Early clinical trials have demonstrated the overall safety and efficacy of NK cell-based therapeutic approaches, such as the use of antibodies or cytokines to enhance the NK cell function, the generation of CAR-modified NK cells or the adoptive transfer of NK cells (or their precursors) from haploidentical donors in treating AML (22, 31). Still, the effort to devise new methods of generating more feasible clinical scales of NK cells is ongoing. Recent technology is exploiting active molecules/cytokine delivery, imaging, and physicochemical properties of nanoparticles to overcome the challenges of NK cell cancer immunotherapy (32).

### Role of NK Cells in Treating Myeloid Malignancies

Early evidence showed that NK cells could kill AML cells, illustrated by the absence of relapsed AML disease after hematopoietic cell transplantation containing alloreactive NK cells (3, 33). High IFN-γ-producing capacity of NK cells was associated with improved anti-tumor immunity (34). In AML, the antileukemic activity of NK cells negatively reflects on disease progression, with NK cells being suppressed at diagnosis, restored at remission, and suppressed again at relapse. Similarly, in CML, the number of NK cells decreases with disease progression, respond less to stimuli, and exhibit reduced cytolytic activity (35). CML patients in complete remission (CR) with a deep molecular response to TKIs have restored NK cell cytolytic activity (36). Furthermore, a high percentage of NK cells at TKI discontinuation was associated with improved long-term outcomes. Comparatively, higher cytolytic activity of NK cells in AML predicts an improved long-term outcome in patients at diagnosis and in remission (37, 38). Therefore, enhancing the cytotoxic activity of NK cells in myeloid malignancies plays a significant role in counteracting disease progression.

## Impaired NK Cell Function in the Tumor Microenvironment

**Figure 1:**
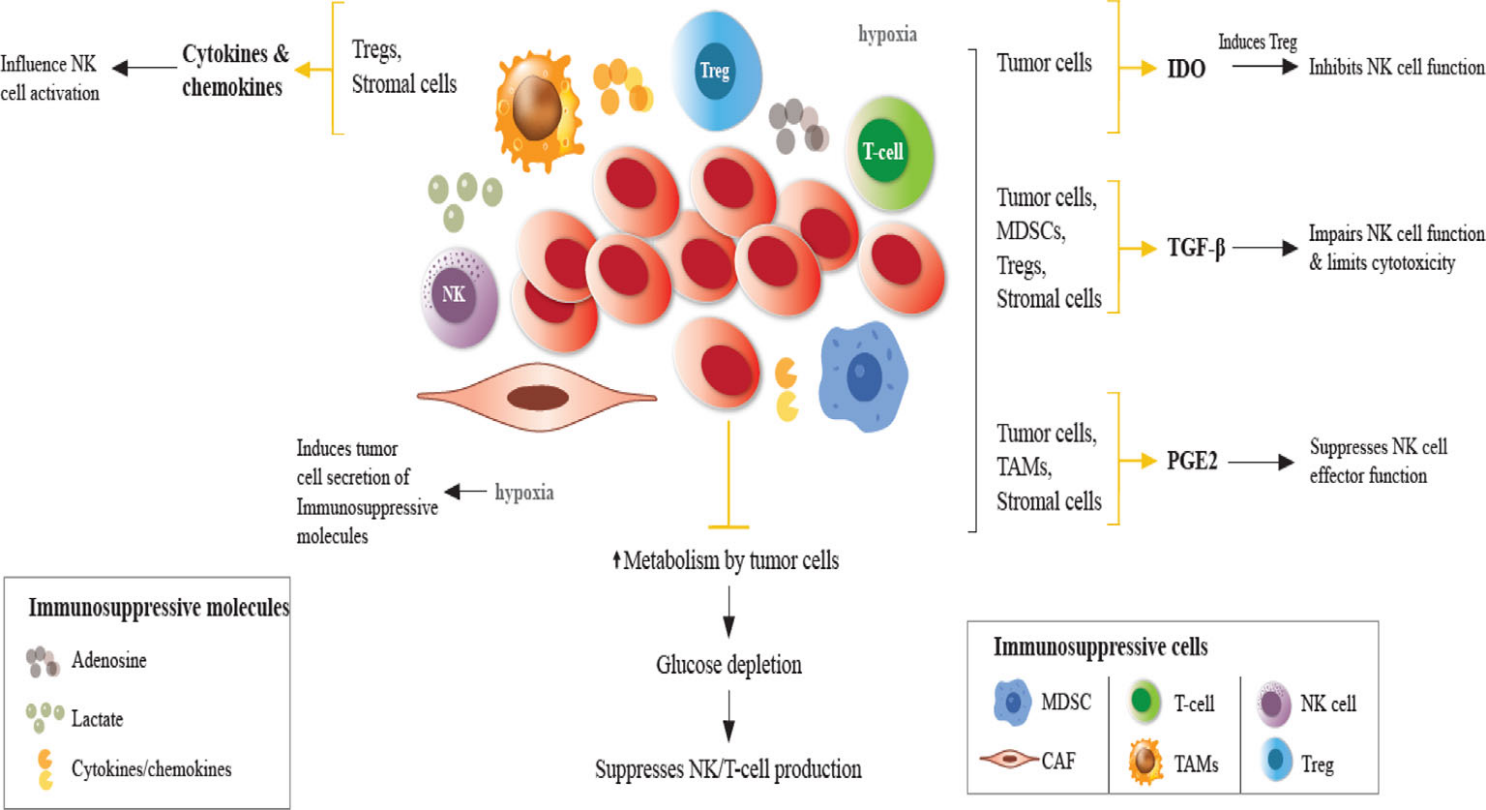
Impaired NK cell function in the TME. In the TME, immunosuppressive cells and molecules, in the presence of hypoxia and glucose depletion negatively regulate the maturation, proliferation, activation, and effector function of NK cells. Hypoxia induces tumor cells to secrete immunosuppressive molecules, such as TGF-β, IL-10, VEGF, galectins, and CC-chemokine ligands, contributing to the accumulation of TAMs, Tregs, MDSCs, which suppress DCs, T and NK cells. Under hypoxic conditions, NK cells fail to upregulate the expression of activating receptors. Cytokines/chemokines produced by Tregs and stromal cells influence NK cell activity. Tumor cells directly exert immunosuppressive effects on NK cells through the secretion of soluble factors. TGF-β production by tumor cells, Tregs, MDSCs, and other stromal cells impairs NK cell function. IDO induces Treg which inhibits NK cell function. IDO expression induces growth arrest of NK cells and promotes tumor progression. PGE2 production in the TME suppresses the effector function of NK cells. Adenosine secretion by tumor cells and lactate accumulation reduces NK cell cytotoxicity promoting immunosuppression by MDSCs, Tregs and TAMs. TGF-β, transforming growth factor-β; PGE2, prostaglandin E2; IDO, indoleamine 2,3 dioxygenase; NKG2D, natural killer group 2D; MDSCs, myeloid-derived suppressor cells; Tregs, regulatory T cells (Tregs); TAMs, tumor-associated macrophages.

The TME plays a critical role in NK-AML recognition. AML cells create an immunosuppressive microenvironment that results in decreased NK cell function, promoting immune escape. As stated earlier, several mechanisms are implicated in the impaired function of NK cells in the TME (**Figure 1**). Modulation of the NK receptor repertoire inhibits NK cell activity in the TME (3). Leukemia cells defectively express ligands for NK cell activating/inhibitory receptors altering NK cell antileukemia response, i.e. decreased expression of NKG2DLs on AML cells caused by aberrant epigenetic mutations renders AML cells resistant to NK cell killing (39, 40).

During AML development, the repertoire of NK cells is modified, reducing the expression of activating receptors, NKG2D, DNAX accessory molecule-1 (DNAM-1), natural cytotoxicity triggering receptor 3 (NKp30), NKp46 (41), and increasing that of inhibitory receptors, killer Ig-like receptors (KIRs), and natural killer group 2A (NKG2A). Moreover, the expression of the ligands of the activating receptors is also modulated (3). Ligands for NKG2D-activating receptor (NKG2DL) can be regulated by DNA methylation (39), reducing the level of expression of NKG2D on the surface of AML cells or is released as soluble activating ligands into the TME, promoting ligand degradation and loss of NK cell cytotoxicity capacity (3, 42).

A study has shown that leukemic blasts can avoid NK cell recognition by decreasing the expression levels of ligands for natural cytotoxicity receptors (NCRs) and natural killer group 2 member D (NKG2D) (35). The down-regulation of ligands for DNAM-1 on the leukemic cell surface promotes resistance to NK cell targeting (43). DNAM-1 receptor has a central role in NK cell-mediated killing of myelodysplastic syndrome (MDS) blasts (44). Additionally, NKG2D and DNAM-1 receptors as well as NCRs play key roles in targeting AML, CML, and acute lymphoblastic leukemia (ALL) blasts (45). Furthermore, NK cell abnormalities, due to interactions with tumor cells or with suppressive immune cells, can favor the escape from immune surveillance in myeloid malignancies (34).

Functional NK cell maturation is defined by differential expression of several markers, including CD56, CD16, CD57, KIR, and NKG2A. In humans, NK cells are divided into two subtypes; CD56^bright^CD16^dim^ and CD56^dim^CD16^bright^, which differ in their homing properties (46). Early study by Lanier and colleagues suggested that the CD56^dim^ NK-cell subset is derived directly from the CD56^bright^ NK subset (47), similarly supported by more recent studies (48–50). Romagnani and colleagues further hypothesized that the differentiation of NK cells corresponded to sequential steps and that secondary lymphoid organs were the sites of NK cell final maturation (48).

Mature NK cells with the CD56^dim^CD16a^+^CD57^+^ subset have great cytolytic potential against tumor target cells and are predominant in peripheral blood (PB). The CD56^bright^CD16a^−^CD57^−^ subset in immature NK cells has high cytokine-producing potential and is more abundant in secondary lymphoid organs (51). CD56^dim^ expresses high levels of cytolytic granules, containing perforin and granzymes that are poorly expressed in CD56^bright^ cells (52). Recently, it has been shown that the cytolytic CD56^dim^ cells produce high levels of IFN‐γ and other cytokines upon receptor‐induced NK cell triggering (53). Moreover, CD56^dim^ NK cells abundantly express CD16 or the low-affinity Fcγ receptor IIIA, which can bind to the Fc region of immunoglobulin. The receptor-ligand binding results in antibody-dependent cellular cytotoxicity (ADCC) which is a dominant component of effective antitumor activity (54).

Defective maturation of NK cells is implicated in chronic viral infections and cancer progression. Multiple lineages of tumors interrupt NK cell functional maturation and impair the antitumor capacity of NK cells (55, 56). Recently, a study by Khaznadar et al. showed that the presence of hypomature NK cells and reduced perforin expression in hypermature NK cells is reflective of defective NK cell maturation (37). In AML, elevated levels of miR-29b reduce the cytotoxic ability of hypermature NK cells, indicating a lesser degree of recognition of AML cells and consequently, increasing the number of hypomature cells (35, 57). MicroRNA miR-29b is a regulator of T-bet and EOMES transcription factors that are essential for terminal NK cell differentiation (57). Upregulation of miR-29b downregulates the expression of T-bet resulting in low perforin levels in mature NK cells, thereby reducing NK cytolytic ability and damage to the development of intermediate NK cells that require expression of both T-bet and EOMES. Therefore, both factors play a role in NK cell generation (58).

Chretien and colleagues reported that in myeloid malignancies, NK cell maturation was perturbed by loss of immature NK cells accompanied by an increase in mature NK cells in the PB of AML patients (59). The study further divided AML patients into three distinct groups based on NK maturation profiles. It demonstrated that AML patients with hypomaturation profiles had reduced relapse-free and overall survival, suggesting that disease-related alterations in NK maturation affected patient outcomes (60).

Three different NK cell maturation stages are reported in AML: hypomaturation (CD56bright/dim KIRs− CD57−), intermediate (CD56dim KIR−/+ CD57−/+) and hypermaturation (CD56dim KIRs+ CD57+) (3). In AML patients at first remission, an increased percentage of immature (CD56^bright^) NK cells was observed, resulting from reconstitution of NK cells after intense chemotherapy (35). In CML, differentiation of NK cells was observed with TKI dasatinib treatment. Upon achieving molecular response, an increased proportion of mature cytolytic NK cells (CD57^+^CD62L^−^) were seen, indicating restoration of NK cell function (36).

Immune checkpoint molecules maintain adequate immune response and play a critical role in self-tolerance and avoidance of cell lysis. The immune checkpoints programmed death 1 (PD-1), TIM3 and TIGIT regulate NK cell activity. PD-1, TIM3 and TIGIT, expressed on NK cell surface recognize their ligands, PD-L1, Gal-9, or CD112/CD155, respectively, expressed on the cell surface of AML cells. As a result, inhibitory signaling occurs in activating pathways involved in NK cell regulation, PI3K, ERK, and PKCΘ, promoting tumor immune escape (61, 62).

High CD200 expression on AML cells has been shown to inhibit antitumor response in AML patients and therefore, is associated with poor overall survival. Coles et al. demonstrated that upregulation of CD200 expression on AML cells suppressed NK cell cytolytic activity and influenced leukemia cell escape from NK cell-mediated lysis (40, 63). As indicated, decreased levels of NKG2D and NCRs, have been reported in patients with AML. A study showed that the overall NCR expression was lower in patients with high CD200 expression on AML blasts, which resulted in CD200-mediated NK cell suppression and contributed to decreased leukemic cell recognition by NK cells (63).

CD200-CD200R interaction on NK cell surface results in immunosuppressive effects that influence responses to pathogens, autoimmunity, transplant tolerance, and cancer surveillance. Targeting CD200 on leukemia blasts presents an insightful strategy to reduce relapse rates and improve outcomes in AML patients (64, 65). A phase I study investigated the therapeutic use of samalizumab (ALXN6000) as a CD200-CD200R immune checkpoint inhibitor in chronic lymphocytic leukemia (CLL) and multiple myeloma. Results showed favorable safety and reduced tumor burden in a majority of patients with advanced CLL with samalizumab treatment (66). Similarly, Herbrich and colleagues identified CD200 as a stem cell-specific immunomodulatory target that aids in establishing an immunosuppressive microenvironment by significantly suppressing cytokine secretion in response to AML stem cell activity (67). Direct contact between AML cells and NK cells, high expression of CD200 on AML cells, soluble NKG2DLs in the sera and suppressive TME are factors that lead to defective receptor expression changes (40).

## Immunosuppressive Factors in the Tumor Microenvironment

### Bone Marrow Microenvironment in Leukemia

In both AML and CML, the bone marrow (BM) microenvironment promotes leukemogenesis through interaction with leukemia stem cells which alter the microenvironment making it hostile for normal hematopoiesis. Targeting these interactions may provide an ample opportunity to treat leukemia more effectively (68). Leukemia cells interfere with the function of the hematopoietic niche and remodel the niche into a favorable environment for expansion or leukaemic transformation (69). Direct cell-to-cell interactions between AML cells and mesenchymal stromal cells in the TME affect the susceptibility of AML cells to NK cells (70).

In the BM, AML cells as well as their neighboring stromal cells, normal hematopoietic cells, and infiltrating immunocompetent cells produce survival and growth-regulatory chemokines and express a wide range of chemokine receptors (71). CCL and CXCL chemokines are the two major chemokine subclasses, which interact with CCR and CXCR membrane receptors, respectively (72). Several cytokines, chemokines and soluble factors have been implicated in the AML/CML BM niche bidirectional crosstalk including CXCR2, CXCR4, IL6R, LFA, VLA4, RANK and FAT/CD36 (73). Bruserud et al. further classified AML patients in three distinct subsets according to chemokine responsiveness and chemokine release profile: CCL2-4/CXCL1/8, CCL5/CXCL9-11, and CCL13/17/22/24/CXCL5 (72).

The tumor microenvironment (TME) and cancer cells exchange signals through a mechanistic process involving soluble factors, such as signaling molecules, and microvesicles. Among soluble factors, chemokines, which induce chemotaxis of target cells, and cytokines play important roles. CXCL12 produced by osteoblasts in BM serves as a ligand for CXCR4 (74, 75). Leukemia stem cells which are also attracted by CXCL12, alter the niche and secrete another cytokine, stem cell factor or KIT ligand, which contributes to the disruption of normal HSC function (76, 77). IL-6 is another cytokine implicated in promoting resistance to chemotherapy in epithelial cancers and hematological stroma-mediated drug resistance (78).

Growth factors are reported to play a role in leukemia-microenvironment crosstalk. TGF-β induces quiescence of HSCs in the niche (79). However, leukemia cells are resistant to TGF-β inhibition despite increased production of TGF-β by BM stromal cells (80). Basic fibroblast growth factor (bFGF) and VEGF are said to promote leukemia development and BM-mediated resistance to chemotherapy (81).

AML blasts induce activation of the vascular endothelium through secretion of inflammatory cytokines, such as TNF-α and IL-10. These induce expression of cell surface molecules on endothelial cells, including ICAM-1, VCAM-1, and E-selectin, promoting adhesion of leukemia blasts to the endothelium (82). Hatfield et al. demonstrated that endothelial cells provide direct support to enhance leukemic proliferation (83). Moreover, interactions between leukemic cells and BM stromal cells induce chemotherapy resistance in AML (84, 85). Various strategies to limit the microenvironment-leukemia crosstalk have been investigated i.e., CXCR4/CXCL12 inhibitors (74, 86), TGF-β neutralizing antibodies (87), and blockage of IL-6 with mAbs (88).

The BM microenvironment also contributes to leukemic cell survival through microvesicles such as exosomes. The TME and tumor cells interact *via* exosomes which are responsible for trafficking proteins and microRNAs that can promote proliferation, metastasis, and apoptosis (89). Additionally, exosomes can suppress hematopoiesis in AML (90). Hong et al. hypothesized that elevated levels of exosomes isolated from pre-therapy plasma of refractory/relapsed AML interfered with anti-leukemia functions of immune cells. The study further showed that AML exosomes could reprogram NK-92 cells, interfering with their anti-leukemia functions and reducing the therapeutic potential of adoptive cell transfers (91). Recently, Dai et al. proposed that the proportion of NK cells in the BM of AML could predict patient prognosis, with a lower proportion of NK cells associated with worse prognosis (92).

AML cells exist under hypoxic conditions in the BM. A hypoxic microenvironment has pivotal prosurvival effects on AML cells through activation of the PI3K-Akt-mTOR pathway and Pim-1 expression (93). Also, leukemia cells actively reduce glucose utilization by healthy cells to increase their bioavailability, resulting in glucose depletion (94). Reactive oxygen species have also been shown to alter NK cell function, affecting antitumor immune response and promoting immune escape within the TME (95–97). In cancer cells obtained from CML patients, reactive oxygen species were implicated in NK cell dysfunction, of which NK cell cytotoxic activity could be restored with catalase suppressive effects of the TME (95, 97).

### TME Suppressive Factors

The TME is a major impediment in enhancing the anti-tumor effects of NK cells. As a complex network, it comprises TAMs, MDSCs, Tregs, regulatory γδT cells, soluble factors, the extracellular matrix, and suppressive molecules expressed on tumor cells (24, 98–101). Tumor cells evade immune escape by establishing an immunosuppressive microenvironment, promoting tumor progression and metastasis (102). Tumor-infiltrating NK cells exhibit poor cytotoxic capacity, accompanied by downregulation of activating receptors, upregulation of inhibitory receptors, and upregulation of immune checkpoint receptors, compared with NK cells from healthy tissues (103). The development of novel approaches such as monoclonal antibodies (mAbs) to block inhibitory receptors enhances NK cell function and its anti-tumor capacity (104). Despite efforts to combat immunosuppressive effects, multiple factors in the TME still limit NK cell function.

Hypoxia, low glucose concentration, cytokines, tumor-derived metabolites, such as adenosine and lactate, tumor cell-derived factors such as IL-6, IL-10, TGF-β, IDO, prostaglandin E_2_ (PGE_2_), and vascular endothelial growth factor (VEGF) (105, 106), including co-inhibitory molecules in the TME such as tryptophan catabolites, dickkopf-related protein 2 (DKK2), soluble HLA-G, soluble NKG2D ligands, and galactin-3 (soluble inhibitory receptor for NKp30) inhibit NK cell activity (107).

In solid malignancies, tumor growth promotes the expansion of immunosuppressive cells, including Tregs, MDSCs, and TAMs. Chemokines, such as C–X–C motif chemokine ligand 8 (CXCL8) or C–C motif chemokine ligand 2 (CCL2) secreted by tumor cells also promote the accumulation of immunosuppressive cells at the tumor site. Through production of TGF-β and IL-10, or *via* direct cell-to-cell interaction, immunosuppressive cells inhibit intratumoral NK cell cytotoxicity (108–110). Tregs directly inhibit NK cell cytolytic function through TGF-β production and downregulate NKG2D expression and NKp30 through membrane-bound TGF-β (111, 112). Furthermore, MDSCs inhibit NK cell cytotoxicity and cytokine secretion through membrane-bound TGF-β on MDSCs in a cell-contact-dependent manner or dependent on NKp30 on NK cells (24, 113, 114). TGF-β, IDO, nitric oxide, and adenosine (108, 115, 116) have also been reported to contribute to MDSC-mediated NK cell inhibition, resulting in reduced NK cell cytotoxicity, decreased NKG2D or NCRs expression, and reduced IFN-γ production (117). TGFβ inhibition with neutralizing antibodies, ligand traps, small-molecule kinase inhibitors, and antisense oligonucleotides are currently under investigation (118).

Expression of high levels of inhibitory molecules, including PD-L1 or PD-L2, on tumor cells, immunosuppressive cells, antigen-presenting cells, and stromal cells in the TME prevent NK cell activation by binding with their respective inhibitory receptors on NK cells, resulting in NK cell exhaustion and dysfunction (119, 120). Cancer-associated fibroblasts (CAFs) are the major stromal cells that affect the antitumor capacity of NK cells. CAFs secrete IDO or PGE2 that downregulate NKG2D expression and secrete TGF-β to reduce the expression of NKG2D, NKp30, and NKp44 and decreasing perforin/granzyme B release (121–123).

In the TME, NK cell metabolism and antitumor responses are impaired (124) due to nutrient and oxygen deprivation and the release of tumor-derived metabolic end-products such as lactate and adenosine, which impede NK cell effector and cytotoxic function (107, 125, 126). Lactate accumulation in the TME results from metabolic reprogramming of cancer cells, characterized by the use of glucose for glycolytic metabolism against metabolizing *via* oxidative phosphorylation (127). Hypoxia leads to decreased pH in the TME and is associated with poor prognosis and resistance to conventional therapies (128, 129). Parodi et al., recently demonstrated that a hypoxic environment could highly influence NK cell infiltration and its effects on immune-mediated responses within tumor tissues (130). Moreover, hypoxia failed to induce CXCL8, VEGF, and MIF secretion by NK cells suggesting that target-specific translational regulation could shape NK cell response to hypoxia. Similar findings were reported by Velasquez and colleagues (131).

Hypoxia also promotes the catalytic conversion of extracellular adenosine triphosphate to adenosine. Stimulation of NK cells through the adenosine A2A receptors suppresses NK cell maturation and impairs anti-tumor immune responses (132). Similarly, in the presence of adenosine, NK cells generate high IFN-γ production reducing NK cell cytotoxicity. Furthermore, hypoxia downregulates NKp30, NKp44, NKp46, NKG2D, perforin, and granzyme B. Treatment with IL-2 has been shown to restore NK cell cytotoxicity by increasing NKG2D in some hematological malignancies (133). Solocinski and colleagues showed that high-affinity NK cells could resist hypoxia through IL-2-mediated prevention of signal transducer and activator of transcription 3 (STAT3) activation, preserving NK cell function (134). On the contrarily, the function of healthy donor NK cells and NK cells from cancer patients were inhibited under hypoxia (135).

Nitric oxide is a critical player in the TME known to cause damage by metabolic reprogramming and promotion of immunosuppressive phenotypes at low concentrations (136). To date, a complete understanding of the mechanisms by which nitric oxide contributes to tumorigenesis is lacking. However, it is known to play a pivotal role in regulating cancer progression in hematological malignancies (137). Dysregulated S-nitrosylation/denitrosylation has been explored as a common mechanism by which NO signaling reprograms metabolism (138).

NK cells utilize glucose to generate ATP and NADPH required for normal function. In a glucose-deprived TME, metabolic competition impairs NK cell glycolytic activity. Amino acid depletion resulting from increased consumption by tumor cells which also synergizes with tumor-associated cells creates a nutrient-depleted microenvironment (139). Other factors are also known to influence NK cell metabolism. Obesity affects metabolic response following cytokine stimulation. Chronic inflammation and infection cause NK cell exhaustion limiting the anti-tumor/infection potential of NK cells. In hematological malignancies, inflammatory cytokines trigger a reshaping of the microenvironment. Further study is required to understand the negative impact of the TME on NK cell metabolism and cancer progression (119).

Targeting metabolic vulnerabilities in AML remains a challenge due to the magnitude of metabolic adaptation in AML cells compared with solid tumors (94, 140). **Table 1**. Gives a summary of various strategies applicable in clinical practice to overcome immunosuppression in the microenvironment.

**Table 1:** Strategies to circumvent immunosuppressive factors in the TME and their role in restoring NK cell function.

Factors restricting NK cell activity in TME	Strategy	Role in enhancing NK cell function	References
Hypoxia	-Hypoxic-activated prodrugs i.e., Evofosfamide (TH-302), PR-104. -HIF-α inhibitors i.e., EZN-2208 (PEG-SN38), Echinomycin (NSC-13502), L-ascorbic acid, and Acriflavine	-inhibit hypoxia-associated resistance to therapy -induce cell cycle arrest and trigger apoptosis -delay leukemia progression	(141–144)
Low glucose concentration	**-**Metabolic regulators: GLUTS **-**glucose-conjugated methyl ester (NHI-Glc-2)	-regulate glucose influx -reduce lactate production -reduce drug resistance, increase conventional drug efficacy	(145, 146)
Tumor-derived end products i.e., lactate, adenosine	-LDHA inhibitors: Galloflavin -AR-C155858 and Syrosingopine -A2aR antagonist: SCH-58261 (SCH)	-inhibit lactate transport -inhibit monocarboxylate transporters MCT1 or MCT4 to impair leukemic cell proliferation -effective anticancer drug delivery -improve tumor control and delay tumor initiation	(127, 147, 148) (132, 149)
IL-6, IL-10	Cytokine prosurvival factors: Recombinant cytokines (IL-2, IL-12, IL-18, IL-15, IL-21, IFNγ, GM-CSF. -IFN-α	Promote, mediate, and regulate immune response and enhance NK cell expansion and cytolytic activity -promote apoptosis and anti-proliferation.	(27, 150–152)
TGF-β	-Fresolimumab (GC1008) and -TGFβR1 inhibitor Galunisertib (LY2157299) -Cilengitide (NCT00089388 AML clinical trial)	- interrupt TGF-β signaling -prevent NKG2D downregulation -restore NK cell anti-tumor function - inhibit αv integrin-TGF-β axis	(24, 118, 153)
PGE2	-PGE2 inhibitors: ASA, NSAIDs, celecoxib.	-prevent tumor initiation -inhibit cancer progression	(154, 155)
IDO	-Inhibitors of immunosuppressive effects of IDO: Indoximod (NCT02835729)	**-**reverse IDO pathway-mediated suppression	(156)
Chemokines	Mogamulizumab	-modulate chemokine receptor expression -enhance NK cell tumor infiltration and improve therapeutic results	(157, 158)
VEGF	Anti-angiogenic therapy: bevacizumab (Bev)	-delay tumor growth -improve chemotherapy drug delivery -promote vascular normalization, senescence and immune cell recruitment	(159, 160)
NO	NO inhibitor: L-NIL	-restore NK cell effector function -enhance NK-cell–mediated ADCC activity -enhance anti-tumor effects of mAbs	(116, 137)

TME, tumor microenvironment; NK, natural killer; AML, acute myeloid leukemia; TGF-β, transforming growth factor-β; GLUTS, glucose transporters; A2aR, A2a adenosine receptor; VEGF, vascular endothelial growth factor; IDO, indoleamine 2,3-dioxygenase; NO, nitric oxide; PGE2, prostaglandin E2; IFNγ, interferon-γ ; IFNα, interferon-α; GM-CSF, granulocyte-macrophage colony-stimulating factor; HIF-α, hypoxia-inducible factor-α; ADCC, antibody-dependent cellular cytotoxicity; mAbs, monoclonal antibodies.

## Improved Therapeutic NK Cell Immunotherapy

### Adoptive Transfer of Autologous and Haploidentical NK Cells

Autologous NK cells were the earliest major focus of adoptive NK cell therapy in cancer patients as a direct approach to restoring and improving immune function at low risk of graft-versus-host disease (GvHD). However, limited anti-tumor activity was reported against hematological and solid tumors, owing to the inhibitory activity of interactions between inhibitory receptors on autologous NK cells and matched self-major histocompatibility complex (MHC) class I presented on tumor cells. Unfavorably, patients were heavily pretreated before collection and in an immunosuppressive state resulting in reduced anti-tumor capability of the derived NK cells. For this reason, allogeneic NK cell therapy came into focus for hematological malignancies (161).

Currently, HLA-matched allogeneic hematopoietic stem cell transplant (HSCT) remains the only curative approach for high-risk AML. Still, the widespread use of HLA haploidentical (half-matched) or partially matched umbilical cord blood (UCB) grafts is ongoing. It has been reported that 25% of patients requiring an allograft have an HLA-identical sibling, while two-thirds of patients have a suitable HLA-matched unrelated donor. For remaining patients lacking an HLA-matched donor, alternative sources are being employed (162–164). Ruggeri et al. suggested that donor-recipient NK cell alloreactivity resulting mainly from KIR ligand incompatibility had decreased relapse rates and graft rejection in AML patients undergoing haploidentical HSCT (165). This concept was termed “ligand-ligand mismatch” (166). Similar studies supported the importance of NK cell alloreactivity in AML patients undergoing HSCT (167–169). Alloreactive NK cells contribute to the graft-versus-leukemia (GvL) effect, which results from the missed interaction of KIRs on donor NK cells and their ligands, HLA class I molecules, on recipient antigen-presenting cells (170). KIR ligand mismatch has a role in the successful engraftment of cord blood and haploidentical transplants (171).

Investigations regarding adoptive NK cell therapy to augment the GvL effect are continual. Miller et al. demonstrated that infusion of haploidentical NK cells after chemotherapy could induce remission in poor-prognosis AML (25). Similarly, Rubnitz et al. investigated the safety of KIR-mismatched NK cell infusion as post-remission consolidation therapy for children with AML. The study reported sustained CR in the 10 patients treated (172). Conclusively, KIR-ligand mismatch correlated positively with NK cell alloreactivity which was protective against leukemia relapse.

Farag et al. however, reported conflicting findings. KIR ligand incompatibility in unrelated donor HCT lacked significant benefit. Unrelated donor HCT showed that HLA mismatch was associated with higher treatment-related mortality, treatment failure, and overall mortality regardless of the presence or absence of KIR ligand mismatch (173). Similar findings report a lack of advantage for KIR ligand incompatibility in clinical setting (174, 175) with inferior outcomes shown with KIR ligand mismatching for hematological malignancies (176). Tanaka et al. evaluated the effect of KIR ligand mismatch on outcomes of AML and ALL patients in CR after single cord blood transplantation. No difference in outcomes of KIR ligand-compatible and incompatible transplantations in acute leukemia patients was reported (177). In an additional study, GvH directed KIR ligand mismatch was associated with graft failure (178). Furthermore, KIR-ligand mismatch seemed to provoke adverse effects in unrelated donor HSCT with reduced overall survival and increased risk for high-grade acute GVHD (170). The heterogeneity of these findings in improving overall survival without increasing GvHD in patients with CR evokes the need for more trials with the application of high-resolution genotyping and phenotyping to measure KIR and HLA of donor and recipient.

Preparatory guidelines for allogeneic NK cell infusion can enhance NK cell expansion, persistence, and efficacy. Non-myeloablative conditioning chemotherapy is administered to deplete endogenous host lymphocyte populations that may compete with the infused NK cells in patients with solid and hematological malignancies (179). Conditioning chemotherapy can potentiate NK cell function in eliminating disease as it serves to directly deplete tumor cells. However, lymphodepletion is associated with increased toxicity including neutropenia. In AML, non-myeloablative therapy administration before infusion of haploidentical NK cells induces CR outside the HSCT setting (25). Following the promising outcomes from the application of NK cells in haploidentical-HSCT, Locatelli and colleagues suggested that their benefit could be extended to curing high-risk acute leukemia and in elderly patients (17).

Miller et al. investigated the safety and *in vivo* expansion of adoptively transferred haploidentical NK cells in a non-HSCT setting. The study demonstrated that that adoptively transferred NK cells derived from haploidentical related donors could be expanded *in vivo*. Adequate lymphodepletion with high-dose cyclophosphamide and fludarabine followed by IL-2 led to *in vivo* NK cell expansion with persistence of donor NK cells for up to 4 weeks without inducing GvHD (25). An inverse correlation between lymphocyte count and IL-15 after lymphodepletion chemotherapy indicated that successful NK cell expansion was secondary to both clearing of alloreactive recipient cells and IL-15 stimulation of NK cells (25, 180, 181).

Recently, Nguyen et al. designed a study to assess the efficacy of adoptive therapy with haploidentical and KIR–HLA-mismatched NK cells for consolidation therapy of pediatric AML. Adoptive transfer of haploidentical and KIR–HLA-mismatched NK cells did not decrease the cumulative incidence of relapse and did not improve event-free or overall survival in children with intermediate-risk AML. However, the study concluded that during earlier phases of treatment or in combination with other immunotherapies, repeated NK cell infusions could reduce tumor burden and induce remission (182). The lack of benefit or long-term remission resulted from insufficient numbers and limited persistence of alloreactive donor NK cells which affected tumor clearance and enabled the re-emergence of leukemic cell clones in patients with relapse (182).

### Exploiting Alternative Sources of Allogeneic NK Cells

**Umbilical cord blood (UCB)** is a rich source of NK cells. NK cells constitute up to 30% of lymphocytes in UCB and 10% of lymphocytes in PB counterparts. Currently, UCB is regarded as an “off-the-shelf,” allogeneic source of NK cells that is readily obtainable and conveniently stored. UCB-derived NK cells are younger with a stronger proliferation potential compared to PB-derived NK cells. UCB-derived NK cells express CD3^-^CD56^+^, further classified as the less differentiated CD56^bright^ and mature CD56^dim^ NK cells (183). Although the degranulation function on UCB-derived NK cells is identical to PB-derived NK cells, their cytotoxicity against K562 leukemia cells is weaker than those acquired from PB (184). However, when stimulated by cytokine activity, the cytotoxicity of UCB and PB-acquired NK cells is comparable.

The infusion of non-modified or modified UCB-derived NK cells as maintenance therapy after chemotherapy or combined with HSCT or UCB stem cell transplantation has shown encouraging results (185). A notable limitation with UCB-derived NK cell infusion is shortened *in vivo* response duration with a gradual decrease in donor NK cells. Developing strategies to improve the persistence of UCB-derived NK cells and maintain relatively high levels of donor NK cells *in vivo* is imperative (186, 187).

**Stem cell-derived NK** cells have recently come into the limelight as an unlimited source of NK cells owing to their homogenous and clinically scalable manner. Furthermore, stem cell-derived NK cells can be readily genetically modified on a clonal level, providing a platform to produce uniform and consistent NK cells with enhanced activity (188). Most adoptive NK cell therapies involve the use of primary NK cells isolated from PB, UCB and NK-92 cells, which have donor-dependent variability, blood collection delays, and challenges in genetic engineering, making product standardization and multiple-dosing strategies difficult.

NK-92 cells, though from a single source, lack many conventional NK cell markers and, as a transformed cell, must be mitotically inactivated before infusion to prevent uncontrolled proliferation (189). NK cells produced from human pluripotent stem cells, both human embryonic stem cells (hESCs) and induced pluripotent stem cells (iPSCs), are being used as standardized “off-the-shelf” therapies for any patient (15). Matsubara et al. showed that hPSC-derived NK cells exhibited cytotoxic properties and could suppress tumor growth *in vivo* without exogenous IL-2 or IL-15 against the leukemic K562 cell line (190). Furthermore, the derived NK cells had similar morphology and surface marker expressions as those of PB NK cells.

**CAR NK** cells have the benefit of easy feasibility as readily available “off-the-shelf” products allowing for multiple infusions in individuals. NK cells of various origins can be genetically modified using CAR constructs that redirect NK cell specificity against antigens expressed on tumor cells (15). Currently, several CAR-NK cells are being tested in phase I/II clinical trials, mostly targeting CD19 but also other antigens including CD7, CD33, BMCA, and CD22 on hematopoietic malignancies (191). Some CAR-NK cells target metastatic solid tumors expressing tumor-associated antigens like HER2, PSMA, mesothelin, ROBO1, or MUC1 (192). CAR NK cells can be used universally without requiring HLA-matching or prior exposure to tumor-associated antigens. Additionally, they cause little or no GvHD while providing therapeutic effectiveness, enhanced cytotoxicity, innate functionality, and *in vivo* persistence in hematological malignancies (11, 193).

CAR NK cells derived from both UCB or NK cell lines such as NK-92 cells can be used as “off-the-shelf” products (194). A recent “first-in-man” clinical trial targeting CD33 tested the safety of CAR NK-92 cells for relapsed/refractory AML patients (195). The trial showed no obvious clinical efficacy. Although multiple infusions of CD33 CAR-NK-92 appeared safe following salvage chemotherapy in relapsed AML, it did not infer an anti-leukaemic effect (195, 196). Liu et al. reported the significant benefit of “off-the-shelf” CAR NK cells in relapsed/refractory CD19-positive lymphoma and leukemia supporting its application in future trials (193, 197).

Notwithstanding that CAR-NK cells have multiple advantages, still several difficulties such as a hostile TME, loss of targeted antigen, and tumor heterogeneity, need to be addressed to maximize the efficacy of CAR-NK based AML immunotherapy (192). Recently, Kararoudi and colleagues described an approach for gene targeting in NK cells using Cas9/ribonucleoprotein complexes. Targeted gene insertion into a safe-harbor locus *via* homologous repair using CRISPR/Cas9 gene editing in combination with adeno-associated virus (AAV)-mediated gene delivery was used to generate primary CD33 CAR-NK cells with confirmation of enhanced anti-AML activity (198).

**Human NK cell lines** have since long served as attractive sources of adoptive therapy. Eight clonal NK-cell lines have been established over the past 20 years, including NK-92, YTS, NK3.3, and NKL. These commonly used NK cell lines have phenotypic and functional differences (199). Specifically, NK-92 remains the only cell line extensively tested in clinical trials for safety and efficacy assessment. NK-92 expresses a phenotype associated with the CD56^bright^ NK cell subset, while both YTS and NKL appear more CD56^dim^-like. Recently, Yang et al. reported on a novel NK cell line, NK101, which produced higher levels of pro-inflammatory cytokines, IFN-γ and TNF-α, than NK-92 and demonstrated an agile immunostimulatory potential and substantial scalability (51).

NK-92 cells are easily expanded *in vivo* using several manufacturing technologies. The safety and efficiency of NK-92 cells with other cell lines such as engineered CAR NK-92 cell lines in both hematologic and solid malignancies has been investigated (200, 201). NK-92 cells are cytotoxic to leukemia cells both *in vitro* and *in vivo* (202). Although several phase I clinical trials have shown the safety and tolerability of irradiated NK-92 cells (203), results of their clinical benefit are still unsatisfying (204). The phase I clinical trial (NCT00900809) of adoptive transfer of NK-92 cells in refractory/relapsed AML reported no antitumor response post-infusion (205, 206).

### Exploiting NK Cell Memory: Priming and Expansion

#### Adaptive NK Cell Therapy

Adaptive NK cells are naturally occurring cell populations that expand in humans upon human cytomegalovirus (CMV) infection or reactivation and represent an imminent source of therapeutic NK cells (207). Adaptive NK cell therapy can reduce the incidence of AML relapse through the surface expression of the maturation marker CD5r7 and the activating receptor NKG2C (208). Adaptive NK cells have extended durability, are more resistant to immune suppression than canonical NK cells, and exhibit properties of immune memory. A study by merino et al. reported that the expansion of adaptive NK cells using NKG2C-agonist antibodies resulted in increased killing abilities and enhanced cytokine secretion, making adaptive NK cells ideal candidates for adoptive cell transfer in hematologic malignancies (209).

Recently Sarhan et al. showed that a subset of “adaptive’ CD57^+^/NKG2C^+^ NK cells obtained from individuals previously exposed to the human CMV exhibited unique properties of immunological memory, potent mediating of ADCC, and resistance to tumor suppression (169). In patients with refractory AML treated with high-dose cyclophosphamide/fludarabine with “adaptive” iv FATE-NK100 NK cell infusion, *in vivo* persistence and function activity of NK cells were observed, with patients achieving clearance of refractory AML by day 14 (210). FATE-NK100 is an investigational, first-in-class, novel NK cell-mediated therapy comprised of adaptive memory NK cells. It is a highly specialized, functionally distinct subset of activated NK cells expressing the maturation marker CD57. FATE-NK100 is currently under investigation for use in lymphodepleted patients with advanced AML, refractory ovarian cancer (210, 211), advanced solid tumors.

The extended durability, *in vivo* persistence, and potency of ‘adaptive” NK cell immunotherapy presents an exciting antileukemia approach. Cichocki et al. demonstrated that human CMV reactivation in HSCT recipients with leukemia was associated with the expansion of adaptive NK cells and correlated with improved post-HSCT (212). In conclusion, the cultivation and expansion of adaptive NK cells *ex vivo* and *in vivo* is likely to yield promising results.

#### Cytokine-Induced Memory-Like NK Cell Therapy

Cytokine-induced memory-like (CIML) NK cells are an alternative for allogeneic NK cell therapy and are known to exhibit antileukemia functionality. Following stimulation with viruses, haptens, or cytokines, NK cells demonstrate innate memory or memory-like responses (213). The use of pre-activation regimens prior to adoptive transfer has been reported to improve NK cell function and enhance antitumor activity. CIML NK cells are generated *ex vivo* through brief priming with IL-12, IL-15, and IL-18 (214). Other cytokines commonly used to activate NK cells, with or without anti-CD3 stimulation (215), include IL-2 and IL-21 (216). Among these cytokines, IL-2 and IL-15 are drivers of NK cell proliferation and enhance function (217). IL-15 is more selective for NK cell proliferation, while IL-2 is associated with increased NKG2D expression (218, 219). Additionally, IL-18 was shown to stimulate IFN-γ production (220) by NK cells and provide co-stimulatory activation, while IL-21 could enhance NK cell maturation without promoting proliferation (221, 222). When comparing feeder cells employed for expansion and activation, chronic stimulation by K562 cells expressing membrane-bound IL-15 and 4-1BB ligand (223) induced senescence, which was not observed with membrane-bound IL-21 (224).

Human memory-like NK cells have enhanced IFN-γ production and cytotoxicity against leukemia cell lines or primary human AML blasts *in vitro* (27). CIML NK cells exhibit enhanced responses against AML blasts regardless of inhibitory KIR to KIR-ligand interactions. Romee et al. demonstrated that the long-lasting increase in functional capacity afforded by memory-like NK cell differentiation, combined with improved AML recognition, enhanced *in vivo* expansion and antileukemia responses (27).

Currently, a phase II clinical trial (NCT04354025) is recruiting patients to investigate the effectiveness of CIML NK cells in combination with FLAG (fludarabine, cytarabine, GCSF) chemotherapy as a treatment for refractory/relapsed AML (225). In an MHC-compatible setting, memory-like NK cells could persist longer than 2-3 weeks, demonstrating a durability and high functionality profile when stimulated with tumor targets. The ability of memory-like NK cells to overcome persistence barriers in an MHC-compatible setting presents a novel platform for NK therapies for leukemia (213).

**Figure 2:**
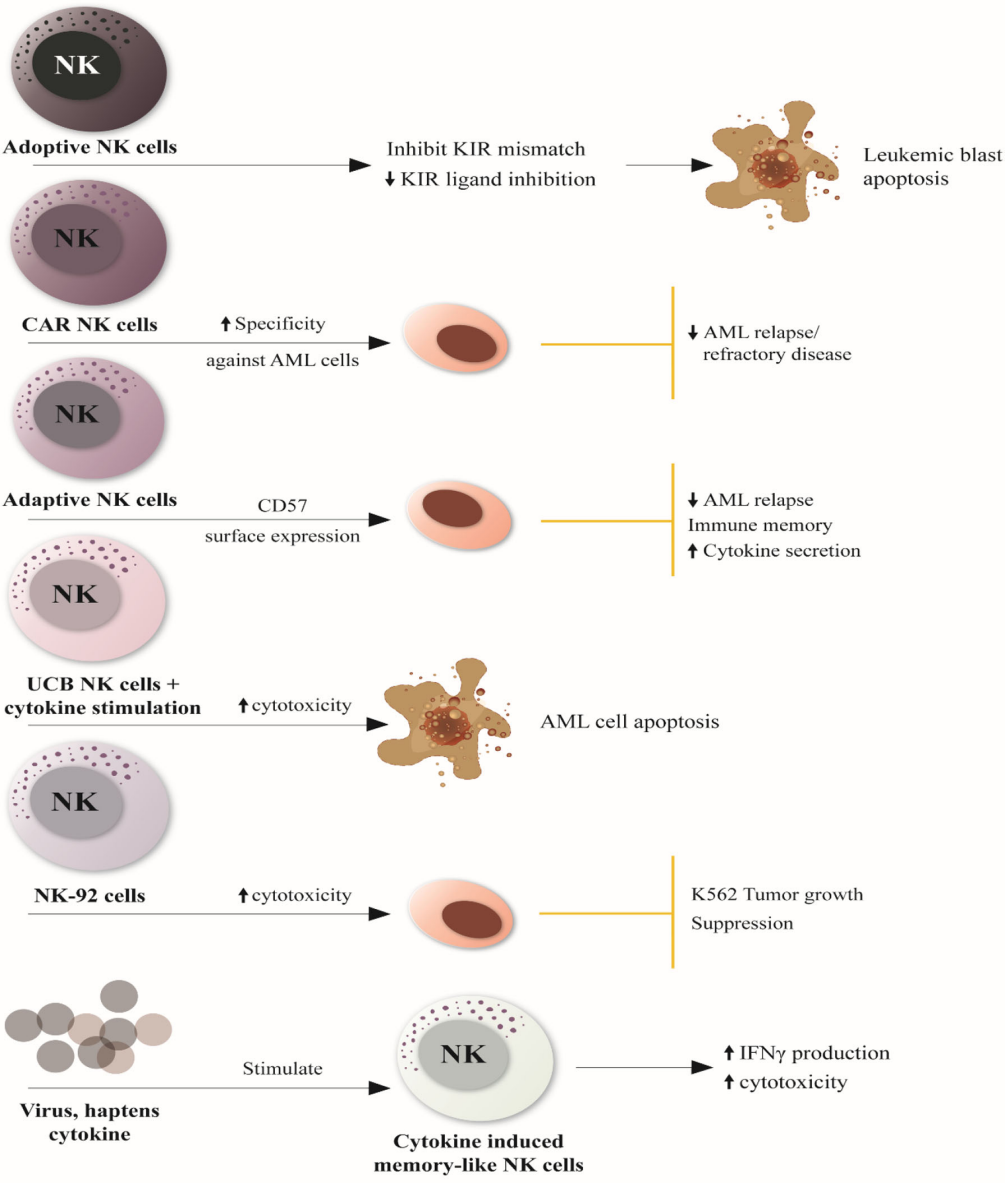
Novel and Improved NK cell-based therapies for AML treatment. 1. Adoptive NK cells can induce leukemic blast apoptosis by inhibiting KIR mismatch and downmodulating KIR ligand inhibitor. 2. CAR NK cells can reduce AML relapse and refractory disease by increasing NK cell specificity against antigens expressed on tumor cells 3. Adaptive NK cells restore immune memory and reduce the relapse of AML through the surface expression of the maturation marker CD57 and the activating receptor NKG2C, resulting in enhanced cytokine secretion. 4. UCB NK cells have increased cytotoxicity against AML cells when stimulated by cytokine activity, promoting cancer cell apoptosis. 5. Irradicated NK-92 cells (NK cell lines) induce tumor growth suppression through increased cytotoxic activity. 6. Various viruses, haptens and cytokines can stimulate innate memory or memory-like responses ‘cytokine-induced memory-like NK cells,” enhancing interferon-γ production and cytotoxicity against leukemia cell lines or primary human AML blasts *in vitro*. NK, natural killer; KIR, killer Ig-like receptor; CAR, chimeric antigen receptor; CD57, cluster of differentiation 57; UCB-NK cell, umbilical cord blood-Natural killer cells; IFN-γ, interferon-gamma.

**Figure 2** illustrates novel and improved therapeutic NK cell therapies explored for the treatment of AML and their specificity in AML.

## Enhancing NK Cell Cytotoxicity Against Myeloid Leukemia

### Immune Checkpoint Inhibition

NK cells have emerged as contributors to the effect of cytotoxic T lymphocyte-associated protein 4 (CTLA4), LAG3, and PD-1 in cancer patients, suggesting that immune checkpoint receptors regulate NK cell activity under pathological conditions (226). The recently identified B7 homolog 3 protein (B7H3) appears to inhibit both T- and NK-cell functions. However, more investigation regarding B7H3, particularly the discovery of its receptors on NK and T cells is needed (227, 228). TIGIT (T cell immunoreceptor with immunoglobulin and ITIM domains), CD96 (TACTILE), and (T-cell immunoglobulin and mucin domain 3) TIM-3, are immune inhibitory receptors that share regulatory functions in both NK and T cells, behaving as immune checkpoint receptors in both cell types (229).

Sialic acid-binding immunoglobulin-like lectins (Siglecs) are immunomodulatory sialic acid-binding receptors expressed on a variety of immune cells including NK cells (230). The most recent members of the inhibitory Siglecs family, Siglecs 7/9, are reported to be expressed on human NK cells. Blocking of Siglec-7/9 increases NK cell cytotoxicity (231). Another inhibitory receptor, CD200R, is expressed on T, B, NK, and myeloid cells (63). Blockade of CD200–CD200R interaction inhibits tumor growth (232). Lastly, CD47 plays an inhibitory role in NK cell-mediated anti-viral or anti-tumor cytotoxicity (233).

Immune checkpoint blockade (ICB) therapies such as anti-PD-1 and anti-CTLA-4 present a rational approach to target checkpoint molecules on NK cells and/or macrophages (234). FDA-approved ICB drugs include the anti-CTLA-4 (ipilimumab), anti-PD-1 (pembrolizumab, nivolumab, and cemiplimab), and anti-PD-L1 (atezolizumab, avelumab, and durvalumab). Antibodies block PD-1, CTLA-4 (235), TIM3, LAG3, and TIGIT counter receptors which could enhance T and NK cell functionality against cancer. In myeloid malignancies, early trial results failed to show the clinical benefit of anti-PD-1 as monotherapy in AML (236) or high-risk MDS (237) and CTLA-4 blockade using ipilimumab in high-risk MDS (238). However, ipilimumab treatment after allogeneic-HSCT showed good responses in 22 patients with various hematological malignancies including 12 AML patients. CR with durability was reported even in refractory disease, including extramedullary AML (239).

Currently, the checkpoint inhibitors, anti-TIM3 (i.e., NCT03489343), anti-LAG3 (i.e., NCT03005782), and anti-TIGIT (e.g., NCT04354246) are under investigation in phase I/II clinical trials. Adverse effects from the use of immune checkpoint inhibitors have been reported. Anti-CTLA-4 agents were associated with relatively high occurrences of colitis and hypophysitis (240). Recombinant cytokine can enhance the efficacy of mAbs. Seo et al. showed that a combination of IL-21 and checkpoint blockade facilitated the effector function of exhausted NK cells in advanced cancer patients with NK-92 class I deficient tumors (241).

### NK Cell Antitumor and Effector Functions

#### Activating and Inhibitory Receptors

Activating and inhibitory surface receptors carry out NK cell functions through a net balance of stimulatory versus suppressive signals resulting in either a response to or tolerance of the target cells (242). In a phenomenon known as *“self-recognition,”* inhibitory receptors detect MHC-I ligands on normal cells, and if present, activating signals are terminated. Upon viral infection or transformation, target cells upregulate stimulatory ligands for activating NK cell receptors such as NKG2D. This interaction induces a level of activating signaling that overwhelms inhibitory signaling through inhibitory receptors such as KIRs and NKG2A, resulting in NK cell cytokine release and cytotoxicity (243). In tumor cells, downregulation of MHC-I ligands of inhibitory receptors results in the loss of inhibitory signaling and in a ”*missing-self*” NK cell activation.

Downregulation of MHC-I and key proteins of the antigen processing and presentation machinery (APM) is a key mechanism of cancer immune evasion (244). Cancer cells can reduce the HLA class I expression under the pressure of T cell surveillance (i.e., to escape T cell response). However, lower HLA-I expression results in reduced engagement of inhibitory KIRs and NKG2A, and consequent increase of tumor cell susceptibility to NK cell activity. In some microenvironmental conditions i.e., in the presence of IFN-γ, tumor cells may recover the original HLA class I expression, increasing their resistance to NK cells (245). In particular, the expression of non-classical HLA-E and HLA-G may play an additional role (246).

Anti-KIR mAbs evaluated in multiple indications, including AML have demonstrated favorable safety profiles (247). A study demonstrated that anti-inhibitory KIR mAb (IPH2101) could enhance antitumor effects of NK cells by blocking the major inhibitory HLA-C-specific KIR (248). In the Effikir trial, Vey et al. reported that Lirilumab as monotherapy for maintenance therapy of elderly patients with AML in first CR did not improve leukemia-free survival (249). However, in combination with anti-CD20 mAbs, anti-KIR enhanced NK cell-mediated, rituximab-dependent cytotoxicity illustrating the potential efficacy of combining a tumor-targeted therapy with an NK-cell agonist (250). Monalizumab, a humanized anti-NKG2A blocking mAb has been shown to increase degranulation and IFN-γ production by unleashing inhibition of NKG2A-expressing cells and promoting T and NK cell effector functions (251, 252).

#### NK Cell-Mediated ADCC

ADCC is a key mechanism of NK cell-mediated antitumor activity. The activating receptor, CD16 or FcγRIII, mainly expressed by the CD56^dim^ NK-cell subset, is necessary for ADCC against IgG coated target cells (227). The tumor-targeting antibody drugs, rituximab, elotuzumab, and cetuximab, exert anti-tumor activity by relying on ADCC as the key mode of action to deplete tumor cells (253). Obinutuzumab, was shown to induce stronger ADCC and direct target cell death *in vitro*. In patients with chronic lymphocytic leukemia, obinutuzumab combined with chemotherapy prolonged progression-free survival (254).

### NK-Cell-Derived Extracellular Vesicles (NKEVs)

NKEVs have been studied for application in cancer therapies and in determining actual NK cell status in patients (255). A recent study demonstrated that NKEVs express MCH I & II suggesting their potential role in MCH class I self-recognition and antigen presentation. Additionally, NKEVs express multiple cytotoxic proteins, such as granulysin, perforin, FasL, and granzyme A and B, as well as molecules that promote NK cell-mediated cytolysis (256). NKEVs also possess regulatory functions and trigger multiple killing mechanisms (257). Cytotoxic effects of NKEVs on tumor cells, including hematological malignancies, were demonstrated in ALL. NKEV treatment elicited endoplasmic reticulum stress that mediated cell death (257).

### NK Cell Engagers

Bispecific or trispecific killer engagers (BiKE, TriKE) are multi-specific antibodies that directly bind NK cells and target cells, triggering NK cell activation and cell-mediated ADCC. The effectiveness of NK cell infusions against leukemic cells is limited by a lack of antigen specificity and *in vivo* expansion. BiKEs and TriKEs create an immunologic synapse or connection between NK cells and myeloid cells (258). In primary AML with impaired NK cell function, TriKE (CD16-IL15-CD33) restored NK cell function and induced NK cell proliferation *in vitro*. Furthermore, 16-15-33 TriKE exhibited superior anti-tumor activity and induced *in vivo* persistence and survival of NK cells (259). In a recent cohort study, Warlick et al. demonstrated that continuous infusion with GTB-3550 TriKE enhanced NK cell proliferation in relapsed AML or high-risk MDS patients and could induce remission. Although no objective responses were reported at initial dose levels, GTB-3550 drove immune activity in humans (260).

### Nanoparticles in Augmenting NK Cell Therapy

Currently, clinical outcomes of NK cell immunotherapy remain unsatisfying owing to an immunosuppressive TME and low NK cell activity. Novel innovations to enhance NK cell function using nanotechnology and nanomedicine are being explored (15, 32, 261). The multifunctionality of nanomaterials and their compatibility with NK cells has shown potential in promoting *ex vivo* expansion of NK cells and NK cell activation. TGF-β inhibition with liposome and nano-emulsion mediated conversion of the immunosuppressive TME (262). Nanoparticles encapsulating IFN-γ were used to improve NK cell homing and infiltration (263). Nano-engagers and nanoparticles have also been applied to improve NK cell activation (264). Ortega-Sanz and colleagues supported the use of magnetic nanoparticles with external magnetic fields to promote the accumulation of cytolytic NK cells *in vivo* (265). Given current evidence, engineered nanoparticles could enhance NK cell activity, proliferation and migration to tumor sites.

## Conclusion

NK cells have widened the treatment paradigm of AML. Despite advancements in chemotherapy, the occurrence of refractory/relapsed disease remains prevalent. Recently, NK cells demonstrated potent anti-AML effects without eliciting detrimental adverse effects, such as GvHD, neurotoxicity, and cytokine release syndrome. However, the effector functions of NK cells are suppressed by the presence of an immunosuppressive microenvironment. The complexity of the TME associated with its heterogeneity and immunosuppressive properties is a major obstacle in achieving optimal anti-tumor activity of NK cells. To achieve desired results from NK cell therapy, it is imperative to develop strategies that enhance NK cell cytotoxicity and improve target cell recognition.

Various strategies including adoptive transfer of cytokine-induced therapies, CAR-modified NK cells, mAbs, targeted nanoparticles and therapies targeting activating and inhibitory receptors in the TME might restore NK cell function and enhance their antitumor activity in AML patients. Overcoming immunosuppressive effects of the TME precipitated by various molecules and factors, through novel strategies that neutralize or block suppressive cytokines and chemokines secreted by tumor cells and inhibit the activity of immunosuppressive and stromal cells will improve future clinical outcomes.

## Author Contributions

NK performed literature research and review and wrote the final manuscript. FZ conceived and revised the manuscript. All authors contributed to the article and approved the submitted version.

## Funding

This project was funded by the National Natural Science Foundation of China [grant numbers: 81270597 and 81770179] for leukemia research.

## Conflict of Interest

The authors declare that the research was conducted in the absence of any commercial or financial relationships that could be construed as a potential conflict of interest.

## Abbreviations

ADCC, antibody-dependent cellular cytotoxicity; AML, acute myeloid leukemia; BiKE, bispecific killer engager; PB, peripheral blood; CAR, chimeric antigen receptor; CR, complete remission(s); CML, chronic myeloid leukemia; DCs, dendritic cells; GvHD, graft-versus-host-disease; HLA, human leucocyte antigen; HSCT, hematopoietic stem cell transplant; IL-10, interleukin-10; IFNγ, interferon-gamma; IDO, indoleamine 2,3 dioxygenase; KIRs, killer Ig-like receptors; mAbs, monoclonal antibodies; MDSCs, myeloid-derived suppressor cells; NK, natural killer; NKEVs, NK-cell-derived extracellular vesicles; NKG2DL, natural killer group 2D ligands; PGE2, prostaglandin 2; TAMs, tumor-associated macrophages; TGF-β, transforming growth factor; TME, tumor microenvironment; Tregs, regulatory T cells; TriKE, trispecific killer engager; TKI, tyrosine kinase inhibitors; UCB, umbilical cord blood.
